# MED7/358: Lessons Learned from Creating a Unique Integrative Medical Website for Web Education

**DOI:** 10.2196/jmir.1.suppl1.e50

**Published:** 1999-09-19

**Authors:** K Dyer, C Thompson

**Affiliations:** ^1^Journey of Hearts: A Healing Place in CyberSpace, LafayetteUSA

**Keywords:** Web-education, Integrative Website, Medical Information, Bereavement Education

## Abstract

**Introduction:**

Although the Internet is becoming a part of daily life for the projected 250 million people who will use it by the year 2000, the medical profession has lagged behind in using this emerging global information network. Physicians, in particular, have been slow to utilize this exciting new medium for patient education. However, others in the health field-pharmaceutical and insurance companies, alternative therapists and herbalists-have rapidly embraced the Internet for providing patient health education as a means of advertising their products. Diverse health-related Websites are now providing web-education to our patients, with much of the information seemingly incomplete, misleading or inaccurate. Our Website is a physician-based Website that addresses the traditionally overlooked areas of grief and loss, providing resources and support.

**Methods:**

In creating the Journey of Hearts Website over the last 1 and1/2 years we have used a variety of methods to design, change, and improve the site. Feedback used included responses from visitors, candid comments from friends, usability study for Sun Microsystem's Website re-design, Website reliability standards from several medical sources, statistical analysis of obtainable site data, combined with our own personal preferences regarding useable Websites.

**Results:**

The three components we found to be most important in designing an effective Website for patient education are: content, ease of navigability and use of artistic elements. The most artistic site cannot replace content and conversely, the most informative site will not be utilized if the content is presented in a dull, uninteresting manner. The most instructional Website will not be utilized if people do not know it exists or cannot locate the site on the Internet, underscoring the importance of 'getting the word out.' Additionally, once at the site, if it is too difficult to navigate, or contains too many of the latest web tricks, visitors are likely to get annoyed and leave, no matter how good the content.

**Discussion:**

The Internet has the potential to be an excellent adjunctive resource for patient web-education and to augment or enhance information given in the ever-shortening primary care office visit. To ensure that the information our patients receive is complete and accurate, physicians need to become the primary providers of medical and health information over the Internet. The Journey of Hearts Website is an evolving, integrative medical Website with a unique design combining medicine, psychiatry, poetry, prose and images-providing medical, non-medical and inspirational resources. With this integrative approach, we have created a site that can be experienced as well as read, is informative and visually therapeutic. In this paper/presentation we will discuss and share the key elements and observations discovered in designing a Website for patient Web-education on grief and loss.

